# Effect of XP-endo Finisher and EDDY activation on post-operative pain after single-visit endodontic treatment: a randomized controlled clinical trial

**DOI:** 10.1038/s41598-025-23628-1

**Published:** 2025-11-04

**Authors:** Yara Fawzy Kotb Elgazzar, Abeer Mostafa Darrag, Dina Ali Mohamed Attia

**Affiliations:** Department of Endodontics, Faculty of dentistry, Tanta University, Tanta, Egypt

**Keywords:** EDDY sonic irrigation, Needle irrigation, Post-operative pain, Single-visit, Xp-endo finisher, Medical research, Signs and symptoms

## Abstract

Advanced irrigation techniques, such as the XP-endo Finisher file and sonic EDDY system, have been introduced to overcome the limitations of conventional needle irrigation by enhancing irrigant penetration and disinfection. However, their influence on post-operative pain remains under investigation. This study aimed to evaluate the effect of these techniques on post-operative pain following single-visit root canal treatment. Thirty patients requiring endodontic treatment for asymptomatic vital maxillary central incisors or canines were enrolled. All procedures were performed in a single visit using the ProTaper Universal rotary system. Irrigation was carried out with 2.5% sodium hypochlorite (NaOCl). Participants were randomly assigned into three equal groups based on the final irrigation protocol: Group I – conventional needle irrigation (control), Group II – XP-endo Finisher file, and Group III – sonic EDDY activation. Post-operative pain was assessed using a Visual Analogue Scale (VAS) at 8, 24, and 48 h, and at 1 and 2 weeks. Data were statistically analyzed with significance set at *P* ≤ 0.05. Pain scores significantly decreased overtime in all groups. While between-group differences were not statistically significant, within-group reductions were significant. Group II exhibited the lowest pain levels, followed by Group III, while Group I reported the highest. Additionally, female participants had significantly higher odds of reporting pain. The XP-endo Finisher and sonic EDDY systems resulted in better pain reduction over time than conventional needle irrigation, with XP-endo Finisher being most effective.

**Clinical relevance: **Advanced irrigation systems may enhance patient comfort and compliance by reducing post-operative pain after endodontic therapy.

**Clinical trial registration:** The study protocol was registered on https://beta.clinicaltrials.gov identifier: NCT05450003 on (08/07/2022).

## Introduction

Effective endodontic treatment success hinges on the comprehensive removal of necrotic or infected pulp tissues, along with the eradication of microorganisms, and the thorough seal of the root canal space^1^.

The conventional method of employing a needle and syringe for delivering the irrigating solution to the root canal system for debridement has been widely adopted and is still commonly utilized in practice^2^. Nevertheless, research has demonstrated that the flushing action provided by syringe irrigation is insufficient, primarily since the irrigating solution reaches only 1 mm past the tip of the needle^1^. Therefore, various delivery and agitation systems have been introduced to improve the irrigation penetration and effectiveness^1,2^.

The XP-endo Finisher file is a novel rotary instrument designed to be utilized following root canal instrumentation, serving as a finishing step to enhance the penetration of irrigants and improve the cleaning and disinfection of the root canal^2^. It is made from a special nickel-titanium (NiTi) MaxWire alloy which exhibits different phases of reactivity at varying temperature levels. At room temperature, it operates in the soft and ductile Martensite (M-phase), allowing for easy deformation. However, when subjected to the elevated temperatures within the root canal, it transitions to the strong and hard Austenite (A-phase)^3^. Hence, when inserted into the canal, it demonstrates the shape memory effect, transitioning from the M-phase to the A-phase, and displays super elasticity during canal preparation^4^.

EDDY is a recently developed sonic device powered by an air scaler operating at a frequency of approximately 5000–6000 Hz. It generates high amplitude oscillating motion in the moving tip through rapid vibration, facilitating enhanced penetration of irrigants and fostering improved cleaning efficacy^5^. It exhibited promising outcomes comparable to those observed with passive ultrasonic irrigation thanks to its high-frequency vibration^6^.

Single-visit root canal treatment offers various benefits, such as a decreased risk of flare-ups, fewer required procedures, and elimination of potential inter-appointment leakage through temporary restorations^7^. However, the primary concern associated with single-visit endodontics is postoperative pain^7,8^.

Postoperative pain commonly arises following root canal treatment, resulting from acute inflammation in the periapical tissues, which can be initiated by various causative factors^8^. VAS has been frequently employed in research examining pain experienced during or following root canal procedures since it provides simple and valid results^8,9^. The VAS intensity rating consists of a 100-mm length line with two end points expressed as no pain and worst pain^9^.

This study aimed to evaluate the effect of Xp-endo Finisher file and sonic EDDY activation on post-operative pain compared to conventional needle irrigation following single-visit root canal treatment.

The null hypothesis of this clinical study was that there would be no difference between Xp-endo Finisher file and EDDY on post-operative pain reduction following single-visit endodontic treatment.

### Aim of the study

The aim of this study was to evaluate the effect of different root canal irrigation techniques (XP-endo Finisher file and sonic EDDY system) on post-operative pain clinically using VAS.

## Materials and methods

### Study design

All methods were carried out in accordance with relevant guidelines and regulations. This randomized clinical trial has been written according to Consolidated Standards of Reporting Trials (CONSORT) guidelines.

### Study setting

This study was conducted on outpatients who presented to the clinic of Endodontic Department, Faculty of Dentistry, Tanta University.

### Sample size calculation

The sample size calculation for this study was based on the findings of Gündoğar et al.^8^, who evaluated post-operative pain following different irrigation activation techniques using the VAS scale. In their study, the response within each subject group was normally distributed with a standard deviation of 1.03. Assuming a true difference of 1.8 between the means of group III and group I, it was estimated that 6 subjects per group would be required to reject the null hypothesis (equal group means) with a power of 0.8 at a Type I error probability of 0.05. Using PS: Power and Sample Size Calculation software and the effect size reported in their work, it was calculated that a minimum of 24 patients would be needed to achieve 80% statistical power at a 95% confidence interval. To compensate for possible dropouts and to enhance statistical reliability, the total sample size was increased to 30 patients.

### Patient selection

For this study, a cohort of thirty individuals aged 18 to 50 in need of endodontic treatment for either maxillary central incisors or canines were chosen.

### Inclusion and exclusion criteria

#### Inclusion criteria

- Patients not using analgesic or sedative medications prior to root canal therapy.
- Asymptomatic vital teeth necessitating root canal therapy.
- Teeth lacking any anatomical anomalies.

#### Exclusion criteria

- Medically compromised patients.
- Pregnant or lactating patients.
- Immunocompromised patients.
- calcified canals.
- Cases with acute apical periodontitis, acute apical abscess and weeping canals.
- Teeth with periapical lesions.

#### Diagnostic standards for case selection

All included cases were permanent anterior teeth diagnosed with asymptomatic irreversible pulpitis in accordance with the American Association of Endodontists (AAE) diagnostic terminology. The diagnosis was based on a combination of patient history, clinical examination, vitality testing, and radiographic assessment.

##### Clinical criteria

Teeth presented with deep caries or mechanical pulp exposure but exhibited no history of spontaneous pain. Patients reported no sensitivity to percussion or palpation, and there were no clinical signs of swelling or sinus tract.

##### Vitality testing

Pulp status was evaluated using a cold test (Endo Ice spray − 50o, Maquira, Maringá, Brazil.). A tooth was considered vital if it responded positively to cold tests, with a normal or non-lingering response but pulp exposed/deeply inflamed.

##### Radiographic criteria

Preoperative periapical radiographs demonstrated intact lamina dura with no evidence of periapical radiolucency or other apical pathology.

##### Baseline pain and analgesic use

Patients were asked about recent analgesic or sedative intake. Subjects who had taken any analgesics within 48 h prior to treatment were excluded. A preoperative VAS was recorded, and only patients reporting no baseline pain (VAS = 0) were included.

#### Group assignment

Patients were randomly allocated using a computerized random allocation program. (Random Allocation Software Version1.0.) into three equal groups (*n* = 10).

- Group 1: Conventional needle syringe (Ultra- dent, South Jordan, UT.) irrigation group (control group).
- Group 2: XP-endo Finisher (FKG Dentaire, La Chauxde -Fonds, Switzerland.) activation group.
- Group 3: EDDY Sonic activation (VDW GmbH, Munich, Germany.) group.

#### Randomization, concealment and blinding

Patients were provided with detailed information about the nature and purpose of the study and provided written consent prior to the initiation of treatment. Thirty patients who provided consent were randomly assigned to one of the three treatment groups, determined by the irrigation system. The randomization and concealment process were handled with the aid of an independent, trained investigator not involved in the study handled. Random sequence generation was achieved using a computer random allocation program with an allocation ratio 1:1:1 and concealed from the operator using the sequentially numbered opaque sealed envelope (SNOSE) technique. Subsequently, a sealed envelope containing instructions specifying the utilization of either conventional needle syringe irrigation, XP-endo Finisher file or EDDY sonic activation was selected after preparation and just prior to final irrigant protocol. The patients were not aware of the irrigation technique they received. Hence, this was a single-blinded trial.

#### Treatment procedure

During the initial visit, a thorough case history was acquired, followed by a detailed clinical examination. Subsequently, a preoperative digital periapical radiograph was captured using a digital intraoral sensor to evaluate the condition of the canal and periapical tissues. Local anesthesia was administered and treated tooth was isolated using rubber dam. After access cavity preparation, WL was established with the assistance of an apex locator and subsequently confirmed radiographically to be 1 mm shorter than the radiographic apex.

#### Root Canal Preparation protocol

The apical portion was first scouted and patency confirmed with a size #25 K-file; this file size represented the baseline apical diameter before rotary instrumentation. Root canals were prepared using the ProTaper Universal rotary system (Dentsply Maillefer, Ballaigues, Switzerland) with a controlled speed of 300 rpm and a torque setting of 2.0 Ncm, as recommended by the manufacturer. The sequence included shaping files SX (19/0.04), S1 (18/0.02), and S2 (20/0.04) for coronal and mid-root enlargement, followed by finishing files F1 (20/0.07), F2 (25/0.08), F3 (30/0.09), and F4 (40/0.06) for apical enlargement.

Instrumentation was performed in a crown-down technique, with each file used in a gentle pecking motion with an amplitude of 2–3 mm until resistance was encountered. Each file was applied for approximately 10–15 s per canal, and irrigation with 2.5% NaOCl (Clorox Co, 10th of Ramadan, Egypt.) was performed between each file change. Apical patency was maintained using a size 10 K-file. The total preparation time per canal was 5–7 min, depending on root canal complexity. A total of 2–5 mL of solution was utilized during access cavity preparation and WL determination, followed by an additional 5 mL after each use of a rotary instrument.

Following chemo-mechanical preparation, a pre-randomized sequentially numbered envelope was opened to identify the final protocol of irrigation for each case. The following irrigation protocol was applied:

- In group 1 (Conventional needle syringe irrigation group):

Irrigation was continuously performed using a 30-gauge side-vented needle inserted into the canal without bending, stopping 2 mm short of the working length. The needle was moved up-and-down for 30 s to enhance the flow rate of the irrigant before being left in place for 60 s.

This final irrigation protocol was done in six cycles 30 s each using a total volume of 10 mL of 2.5% NaOCl solution in four cycles and 5 ml of 17% EDTA solution in two cycles. The initial two and final two cycles involved the use of NaOCl, while the two cycles in between utilized EDTA.

- In group 2 (XP-endo Finisher group):

Final irrigation protocol and NaOCl were applied in the same manner as in Group 1.Xp-endo Finisher file was then used after completion of instrumentation for each irrigation cycle. The file was cooled down using a cold spray (Endo Ice spray − 50o, Maquira, Maringá, Brazil.). The file was removed from the plastic tube in a rotating manner with simultaneous lateral movement to maintain its straight alignment. Subsequently, the rotation was halted, and the XP-endo Finisher was inserted into the canal in a straight position. Upon inserting the XP-Endo Finisher file into the canal in its straight form, the access cavity was then filled with irrigant, following the manufacturer’s instructions. Rotation was resumed, and the file was activated for 30 s during each irrigation cycle at 800 rpm and 1 Ncm, using slow and gentle 7–8 mm lengthwise movements to ensure thorough contact with the full length of the canal as recommended by the manufacturer.

- In group 3 (EDDY Sonic Activation group):

In group 3, final irrigation protocol was applied in the same manner as in Group 1. EDDY tip was used after each irrigation cycle. EDDY tip was inserted inside the canal after marking WL and manipulated in a vertical motion within a range of 3 mm, commencing 1 mm from the apical terminus, without exerting pressure following the manufacturer’s instructions.

The canals were then dried using paper point (Dentsply Maillefer, Ballaigues, Switzerland). The canals were obturated with AH Plus sealer (Dentsply DeTrev GmbH, Konstanz Germany) with gutta percha (Dentsply Maillefer, Ballaigues, Switzerland) using cold lateral condensation technique.

The AH Plus sealer was introduced into the root canal using a master gutta-percha cone coating technique to ensure uniform distribution along the canal walls. The sealer was applied by lightly coating the master gutta-percha cone before its insertion into the canal. The cone was then slowly introduced to the working length, allowing the sealer to be evenly spread along the root canal walls. This was followed by cold lateral condensation using standardized accessory gutta-percha cones and a finger spreader to achieve a dense, three-dimensional obturation. Then post-operative radiographic evaluation was done to ensure ideal root canal obturation.

Post-operative periapical radiographs were reviewed by two blinded examiners to assess obturation length, density, and the presence/absence of sealer extrusion. Disagreements were resolved by consensus.

#### Postoperative pain evaluation

Prior to the procedure, the clinician obtained the initial record of patients’ pain levels to confirm their comprehension of pain-related instructions. Subsequently, five additional assessments were conducted at postoperative intervals of 8, 24, 48 h, one week, and two weeks, respectively.

All procedures were performed in the morning (8:00–11:00 AM), and pain assessments were recorded in the morning at each time interval (8, 24, 48 h, one week, and two weeks postoperatively) to maintain consistency in data collection.

Postoperative pain assessment was conducted utilizing VAS, where patients were able to indicate their pain level by placing a mark anywhere along a horizontal line ranging from 0 to 100. Scoring was conducted in accordance with the methodology outlined by Jensen et al.^9^ where:

- No pain where the treated tooth felt normal was marked from 0 to 4 mm and represented by **score 0**.
- Mild, detectible, but not discomforting pain with no need of analgesics was marked from 5 to 44 mm and represented by **score 1**.
- Moderate, discomforting, but tolerable, pain (analgesics when used were effective in relieving pain) was marked from 45 to 74 and represented by **score 2**.
- Severe, difficult to bear pain where analgesics had slight or no effect in pain relief was marked from 75 to 100 mm and represented by **score 3**.

Each patient was provided with a VAS form to carry with them. Patients were kept unaware of the specific irrigation technique they received. They received telephonic reminders to record their pain readings and return the completed form. Only when needed a prescription of 600 mg of Ibuprofen for patients recording score 3 was given under the supervision of the endodontist.

#### Statistical analysis

Statistical analysis was performed using the Statistical Package for Social Sciences (SPSS) version 26 (SPSS Inc., Chicago, USA). Data distribution was assessed for normality using the Shapiro-Wilk test, which indicated a non-normal distribution of pain scores. Consequently, non-parametric tests were used for analysis. The Kruskal-Wallis test was applied to compare pain scores among the three groups, while within-group differences over time were assessed using the Friedman test. To evaluate the effects of time and group on pain scores, repeated ordinal logistic regression with a generalized estimating equation (GEE) approach was employed. Odds ratios (OR) with 95% confidence intervals (CI) were reported to interpret the likelihood of higher pain scores over time. A p-value ≤ 0.05 was considered statistically significant.

## Results

**Fig. 1:**
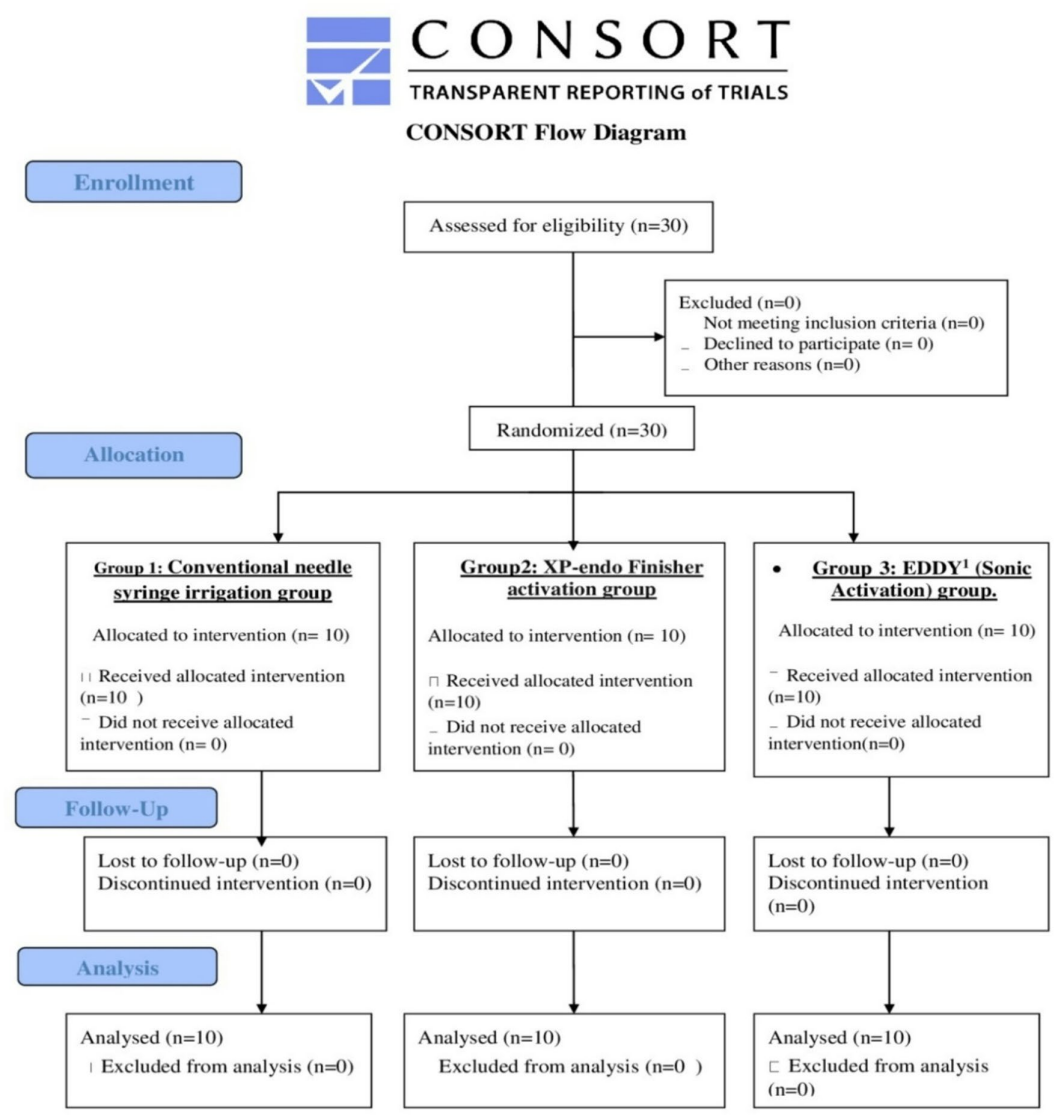
CONSORT flowchart.

There were no dropouts or missing primary outcome data; therefore both intention-to-treat and per-protocol analyses were identical. The flow chart of the patients throughout the study presented in the CONSORT flow diagram (Fig. 1).

### Demographic data

#### Gender

The gender distribution among the three study groups was analyzed using the chi-square test. Group I included 6 males (60%) and 4 females (40%), Group II had 5 males (50%) and 5 females (50%), and Group III consisted of 6 males (60%) and 4 females (40%). The chi-square test revealed no statistically significant difference in gender distribution among the groups (χ² = 0.273, *p* = 0.873), indicating that gender was evenly distributed across all study groups (Table 1).

**Table 1:** Percentage of gender for the tested groups.

Gender	Group I	Group II	Group III	*p*-value
Females	4 (40%)	5 (50%)	4 (40%)	0.873 (ns)
Males	6 (60%)	5 (50%)	6 (60%)	

### Pain intensity at different time intervals

A univariate analysis was conducted to assess the effects of time and group differences on pain scores. Pain scores were evaluated at different time points (8 h, 24 h, 48 h, 1 week, and 2 weeks) across three groups. Between-group differences were not statistically significant, but within-group differences over time were significant (Friedman test: Group I, *p* < 0.001; Group II, *p* = 0.009; Group III, *p* < 0.001). The effect of time was significant across all groups, with all groups reporting a median pain score of 0 by the end of the study period (Table 2).

**Table 2:** Univariate analysis to evaluate time and group effects on pain score.

Time	Pain score	Group I		Group II		Group III		Test statistic§	*p*-value
		No	%	No	%	No	%		
8 h	0	2	20	6	60	3	30	6.26	0.193
	1	5	50	4	40	6	60		
	2	3	30	0	0	1	10		
	Median (IQR)	1	0.75–2.75	0	0–1	1	0–1	5.14	0.076
24 h	0	4	40	6	60	5	50	1	0.95
	1	4	40	3	30	3	30		
	2	2	20	1	10	2	20		
	Median (IQR)	1	0–1.25.25	0	0–1	0.5	0–1.25.25	0.83	0.66
48 h	0	4	40	9	90	6	60	5.46	0.088
	1	6	60	1	10	4	40		
	Median (IQR)	1	0–1	0	0–0	0	0–1	5.27	0.072
1 week	0	7	70	10	100	9	90	4.04	0.28
	1	3	30	0	0	1	10		
	Median (IQR)	0	0–1	0	0–0	0	0–0	3.9	0.142
2 weeks	0	10	100	10	100	10	100		
	Median (IQR)	0	0–0	0	0–0	0	0–0	0	1
Time effect¥		Test = 22.56 *P* < 0.001		Test = 13.41 *P* = 0.009		Test = 19.6 *P* < 0.001			

IQR, interquartile range; §, chi-square with Monte-Carlo method and Kruskal-Walis test; ¥, Freidman test.

Between group differences in pain score across the time were not significant.

Within group differences along the time were significant.

Repeated ordinal logistic regression using the generalized estimating equation approach was applied to predict pain scores over time. Time had a significant effect, showing a 67% decrease in the odds of reporting a higher pain score at each successive time point (OR = 0.33, *p* < 0.001). Group II had significantly lower odds of reporting higher pain scores compared to Group I (OR = 0.09, *p* = 0.02), while Group III had 4.76 times the odds of reporting higher pain compared to Group II. Female participants had significantly higher odds of reporting pain (OR = 4.31, *p* = 0.05) (Table 3).

**Table 3:** Repeated ordinal logistic regression using generalized estimating equation to predict pain score over time.

Parameters	Beta	SE	95% Wald CI		Wald χ2	OR	*p*-value
Thresholds							
Score = 0	−2.54	0.88	−4.27	−0.82	8.34	0.08	0.00
Score = 1	0.28	0.68	−1.06	1.61	0.17	1.32	0.68
Group = 3	−0.84	0.81	−2.43	0.75	1.07	0.43	0.30
Group = 2	−2.40	0.99	−4.33	−0.47	5.92	0.09	0.02*
Group = 1¥	Reference						
Group = 1	0.84	0.81	−0.75	2.43	1.07	2.31	0.30
Group = 2	−1.56	0.81	−3.14	0.02	3.74	0.21	0.05*
Group = 3¥	Reference						
Time	−1.11	0.14	−1.39	−0.83	59.36	0.33	< 0.00*
Female	1.46	0.75	−0.01	2.92	3.81	4.31	0.05*

Beta, regression coefficient; CI, confidence interval; OR, odds ratio; SE, standard error; *, significant p-value at 0.05 level; ^¥^, reference category using different sort.

The odds of a patient in group I reporting a higher pain score was 11 times (OR = 0.09) that of a patient in group II over the 5 measurement times, adjusting for repeated gender.

Group III patients had 4.76 times the odds of reporting a higher pain score than group II patients over the time after adjusting for within-subject correlation and gender.

The effect of time on pain score was significant, whereas the odds of reporting a higher pain score decreased by 67% with each time point (OR = 0.33).

Interaction terms between time and group were introduced in the regression model to assess whether the effect of group on pain score varied over time. The interaction was not statistically significant, indicating that the effect of group on pain score remained stable. Time remained a significant factor, reinforcing the trend of decreasing pain scores. Gender differences in pain perception persisted with higher pain reported in females than males (Table 4).

**Table 4:** Repeated ordinal logistic regression using generalized estimating equation to predict pain score over time with introducing interaction terms.

Parameters	Beta	SE	95% Wald CI		Wald χ2	OR	*p*-value
Thresholds							
Score = 0	−2.57	0.99	−4.50	−0.63	6.73	0.08	0.01
Score = 1	0.26	0.77	−1.25	1.76	0.11	1.30	0.74
Group = 3	−0.91	1.07	−3.01	1.20	0.71	0.40	0.40
Group = 2	−2.38	1.32	−4.97	0.21	3.24	0.09	0.07
Group = 1	Reference						
Group = 3 * time	0.03	0.17	−0.30	0.36	0.03	1.03	0.87
Group = 2 * time	−0.01	0.25	−0.51	0.49	0.00	0.99	0.96
Group = 1 * time	Reference						
Time	−1.12	0.18	−1.47	−0.77	38.35	0.33	< 0.001*
Female	1.46	0.75	−0.01	2.92	3.80	4.31	0.05*

Beta, regression coefficient; CI, confidence interval; OR, odds ratio; SE, standard error; *, significant p-value at 0.05 level.

The interaction between group and time was not significant, implying that the effect of group on pain score did not vary across the time.

Overall, pain scores significantly decreased over time across all groups. Although between-group differences were not significant, within-group changes were observed. Group II had the lowest pain scores, while Group III had higher odds of reporting pain than group II and Group I showed the highest pain scores. The effect of time suggests progressive pain alleviation, with gender differences requiring further investigation.

No sealer extrusion was observed on postoperative radiographs in any of the treatment groups. Therefore, all cases were included in the analysis without the need for subgroup comparison.

## Discussion

Success of endodontic therapy extends beyond the effectiveness of cleaning, shaping, and proper obturation. It also depends on ensuring the patient experiences the least level of discomfort^10^. Therefore, all endeavors are focused on delivering the patient optimal root canal treatment while minimizing or eliminating any post-operative pain. The reasons behind the occurrence and intensity of pain remain unclear as research on pain evaluation is hindered by various limitations together with the multifactorial etiology of post-operative pain^11^.

The current study was structured as a single-blinded parallel randomized controlled clinical trial, recognized as one of the most robust study designs. The incorporation of three key randomization components (random sequence generation, concealment, and implementation) ensured an equal opportunity for all participants to be assigned to any of the study groups. This approach was deemed optimal for achieving a balance of unidentified prognostic factors among participants across the three groups, thereby mitigating potential biases in selection or allocation^12^.

The inclusion and exclusion criteria and the methodological procedures of this study were meticulously implemented to minimize the effect of any additional factors and to induce the least post-operative pain in patients in order to eliminate the confounding effect of various preoperative factors and to precisely investigate the effect of adding XP-endo Finisher file and the new sonic EDDY system to the irrigation protocol on post-operative pain.

The maxillary central incisors and canines were chosen for this study because they have almost wide and straight canals, making apical preparation to ProTaper F4 acceptable. On the contrary, maxillary lateral incisors were excluded because they have more anatomical variation and curvatures in the apical third, as well as thin roots where apical preparation to ProTaper F4 would be overzealous.

Asymptomatic teeth were chosen since preoperative pain serves as a highly reliable predictor of postoperative discomfort. Additionally, strict adherence to an aseptic protocol was upheld to minimize the potential for exacerbation caused by residual microorganisms or the introduction of bacterial contamination^13^.

Furthermore, this study included only patients with no relevant medical history who had not taken pain-relieving medication recently. This selection criteria aimed to ensure that any additional sources of pain or potential drug interactions would not affect the pain experienced because of the endodontic therapy.

The decision to complete the endodontic treatment in a single visit aimed to eliminate the need for intracanal medication, which could potentially contribute to post-operative flare-ups. Additionally, this approach sought to prevent the risk of root canal recontamination and bacterial regrowth associated with prolonged treatment, thereby reducing the likelihood of experiencing pain^14^.

Canals were manually irrigated using syringe irrigation, which is widely favored and prevalent for being simple and easy, offering control over needle depth and irrigant volume. Currently, side-vented tips are utilized for irrigation due to their ability to create a low-intensity jet that directs the irrigant towards the root canal walls, enhancing hydrodynamic movement. This results in increased pressure on the canal walls, facilitating irrigant reflux and displacing debris coronally, thereby minimizing the risk of apical extrusion^15^.

In each group, the needle was inserted to a depth of 2 mm short of the working length. This depth of penetration was chosen based on established safety protocols outlined in previous studies, which recommend inserting needles 1.5–3 mm short of the working length during final irrigation to mitigate debris extrusion. Furthermore, manual agitation of the needle in vertical strokes was conducted, as studies have shown that this technique reduces the extrusion of irrigant and debris into the periapical tissues. Such extrusion is known to contribute to periapical inflammation, postoperative pain, and delayed healing^16^.

Xp-endo Finisher was chosen as several studies revealed that Xp-endo Finisher is more effective than conventional syringe and needle irrigation in eliminating root canal debris^17^ and numerous researchers have shown that the use of Xp-endo Finisher file significantly reduces the number of bacteria^18–20^.

Moreover, the new sonic EDDY activation system was chosen as it showed encouraging outcomes akin to passive ultrasonic irrigation. Thanks to its high-frequency vibration^6^. EDDY system was also shown to achieve better removal of debris and smear layer than the traditional irrigation delivery system^21^.

The cold lateral condensation technique was chosen for its ability to achieve a tight apical seal with minimal stress on the canal walls, which helps reduce post-operative pain. Unlike warm vertical compaction, which may generate apical pressure and extrusion of filling material, lateral condensation provides a more controlled obturation, minimizing periapical irritation. Studies have shown that an effective seal prevents bacterial microleakage, promoting periapical healing and reducing pain intensity after treatment^22^.

Final apical enlargement to ProTaper F4 (40/0.06) was chosen because the included teeth (maxillary central incisors and canines) typically allow safe preparation to this size. This approach standardized apical diameter across cases, facilitated effective irrigant exchange and activation, and allowed proper accommodation of the activation tips^13^. It should be acknowledged that enlargement to size 40/0.06 may influence irrigant dynamics, risk of extrusion, and subsequent postoperative pain; however, in this study safeguards were applied, including the use of side-vented needles positioned 2 mm short of working length, activation with gentle vertical motion without apical pressure, and obturation by lateral condensation to minimize extrusion risk.

Pain intensity assessment was conducted before and after the procedure at intervals of 8, 24, and 48 h, as well as one and two weeks postoperatively. These time points were selected for specific reasons: the 8-hour interval allowed adequate time for the effects of the anesthetic solution to dissipate completely, while the 24 and 48-hour intervals corresponded to the typical peak periods of pain. Additionally, the one and two-week intervals were chosen based on the observation that retrospective pain evaluation in dental patients tends to be more reliable after one to two weeks^23^. Singh et al.^24^, stated that certain patients might still feel pain for up to 7 days following chemo-mechanical preparation, prompting the recording of pain levels at this interval. Finally, two weeks were chosen as a final assessment interval to reassure relief of pain, if present, and the success of endodontic treatment.

All procedures were performed in the morning (8:00–11:00 AM) to minimize variations in pain perception due to circadian rhythms and patient fatigue. Conducting treatment in the morning aligns with higher cortisol levels, which have anti-inflammatory properties and may contribute to reduced post-operative discomfort. Similarly, pain assessments were recorded in the morning at each time interval to ensure consistency and reduce potential biases related to fluctuating pain sensitivity throughout the day. Prior studies show that pain sensitivity and inflammatory mediators follow circadian rhythms and that morning cortisol levels (cortisol awakening response) and other circadian factors can influence pain perception^25–27^.

Presently, NSAIDs stand out as one of the top choices for pain relief in dentistry due to their minimal side effects. Nonetheless, it is advised against prescribing these medications for regular use following single-visit root canal treatment; instead, they should be given as needed. As a result, a prescription of Ibuprofen 600 mg was provided on demand.

Overall, there were no indications of other symptoms or complications such as swelling or paresthesia among the patients. These findings highlight the meticulous care taken to administer an atraumatic treatment protocol, which consequently led to favorable pain outcomes.

The null hypothesis of this study, which proposed that there would be no difference between XP-endo Finisher and EDDY in reducing post-operative pain, was accepted, as no statistically significant differences were observed between the two groups. However, differences in pain records and clinical findings remain clinically relevant and are therefore discussed.

Pain values were higher in the conventional needle group compared to Xp-endo Finisher group. The heightened pain levels associated with conventional needle-syringe irrigation may be due to the positive pressure exerted during irrigation, resulting in the extrusion of a larger volume of debris towards the apex. Alternatively, it could also be elucidated by the inability to fully reach the complete working length, potentially leaving behind pulpal remnants and microbes that may contribute to the documented post-operative pain^28^.

This finding aligns with several systematic reviews and meta-analyses comparing postoperative pain levels between conventional irrigation and activated irrigation techniques. These studies suggest that alternative methods for activating irrigating solutions are recommended to improve their ability to penetrate and spread chemical solutions throughout the complex anatomy of root canals, resulting in improved postoperative pain outcomes. This stands in contrast to conventional irrigation, which may fall short in adhering to certain principles of contemporary endodontics by inadequately disinfecting the entire root canal system and failing to effectively remove debris, ultimately leading unfavorable pain outcomes^23–30^.

The lowest degree of post-operative pain was recorded by Xp-endo Finisher group. This was supported by other studies which provided that Xp-endo Finisher instrument enabled the breaking down of the accumulated hard tissue and its subsequent removal through the flushing action of the needle in accordance with Leoni et al.^31^, Additionally, the XP-Endo Finisher exhibited significant efficacy in accessing difficult-to-reach and unexplored canal regions, resulting in enhanced cleaning and superior elimination of smear layer and bacterial biofilms, as revealed by Bao et al.^20^,, El Naghy et al.^32^,, On top of that Azim et al.^19^, and Alves et al.^33^, demonstrated that the XP-Endo Finisher demonstrated notable effectiveness in decreasing bacterial counts and demonstrated satisfactory disinfection capabilities through Its high flexibility that allows it to expand up to 6 mm in diameter, enabling greater contact with the root canal walls. This adaptability enhances both chemical activation of irrigants and mechanical debridement, leading to improved smear layer and biofilm removal. By increasing canal wall contact and agitation, the XP-Endo Finisher facilitates the dislodgment of residual debris, reducing bacterial load and optimizing disinfection. These combined effects likely contributed to the superior post-operative pain outcomes observed in the XP-Endo Finisher group compared to conventional irrigation methods.

Moreover, according to a study by Ihab et al.^34^, the addition of the Xp-endo Finisher to the irrigation protocol though not resulting in a significant difference in post-operative pain compared to the conventional irrigation technique in necrotic teeth, it showed lower incidence of pain.

This was in disagreement with Hanafy et al.^35^, who noted that XP-endo Finisher resulted in more post-instrumentation pain scores but without a significant difference when compared to other subgroups. This inconsistency might be attributed to differences in the experimental setup and design.

No statistically significant difference was observed between the Xp-endo Finisher and EDDY groups in terms of postoperative pain. Therefore, the null hypothesis was accepted. However, it was noted that the EDDY group exhibited a higher incidence of pain. This could be attributed to the EDDY system’s three-dimensional motion. This complex motion, along with the system’s higher frequency and more flexible tips, may account for the significantly greater apical debris extrusion associated with the EDDY system in accordance with Ince Yusufoglu et al.^5^, as debris and irrigant extrusions recognized as one of the key contributors to the intensity of post-operative pain, has the potential to induce chemical irritation in the periapical area, consequently leading to post-operative pain.

These results were supported by Dos Reis et al.^36^and Uğur Aydın et al.^37^, who found that EDDY group showed a higher amount of debris extruded apically than the other tested systems.

Although there was no statistically significant difference detected between the conventional needle and EDDY groups regarding post-operative pain, the EDDY group showed a lower incidence of pain. This was in agreement with Gündoğar et al.^38^, who compared post-operative pain levels after 8, 24, 48 h and one week of single-visit treatment. Patients subjected to conventional needle irrigation exhibited significantly greater pain severity at the 24-hour interval compared to those treated with EDDY irrigation.

This may be explained by the high cleaning efficiency produced by the EDDY system demonstrating promising results similar to Ultrasonic irrigation activation as reported by Urban et al.^21^, and Swimberghe et al.^39^, Also, better irrigant penetration achieved by the EDDY system allows for better removal of pulp tissue and promotes its disinfection ability as emphasized by Tungsawat et al.^40^.

This was in disagreement with Paixão et al.^41^, who observed that EDDY irrigation is linked to a heightened occurrence of post-operative pain, particularly within the initial 24-hour period, in contrast to manual irrigation.

Following the treatment, post-operative pain peaked after 8 h and gradually decreased over subsequent monitoring periods, nearly disappearing after one week and being completely absent after two weeks. This phenomenon may be ascribed to the possibility of irritation occurring in the periapical region due to endodontic intervention, potentially intensifying or initiating an inflammatory reaction within the periapical tissues. Between 6 and 8 h, polymorphonuclear leukocytes (PMNs) and pain mediators start to migrate toward the affected area, subsequently leading to elevated levels of inflammatory mediators and neuropeptides. The proliferative phase typically commences after 48–72 h, marked by a reduction in the PMN population and the infiltration of macrophages into the wound site, ultimately leading to the recovery of the periapical area^42^.

The study also found that female participants reported higher pain scores compared to males, indicating a significant effect of gender on pain perception. This difference may be attributed to biological and psychological factors, including hormonal variations that influence pain sensitivity and inflammatory responses. Estrogen, for example, has been shown to modulate pain perception, potentially making women more sensitive to post-operative discomfort. Additionally, psychological factors such as anxiety and pain anticipation, which tend to be higher in females, may contribute to the reported differences in pain levels. These findings highlight the importance of considering patient-specific factors when evaluating post-operative pain and suggest that pain management strategies may need to be tailored based on individual patient characteristics^3^.

Although both activation systems (XP-endo Finisher and EDDY) demonstrated lower postoperative pain scores compared to conventional syringe irrigation, these differences did not reach statistical significance. This finding suggests that while activation systems may provide clinical benefits in irrigant penetration and debridement, their measurable impact on pain perception is less pronounced. Postoperative pain is inherently multifactorial, influenced not only by irrigation protocols but also by patient-related factors such as gender, individual pain thresholds, and inflammatory responses, all of which may obscure subtle intergroup differences.

The absence of significant differences may also be attributed to the relatively small sample size. While the trial was powered based on prior studies, a larger population might be required to detect modest differences in pain outcomes across irrigation protocols. Furthermore, it is possible that when all groups receive thorough chemomechanical preparation and effective irrigation, the baseline level of postoperative discomfort is already minimized, reducing the likelihood of large intergroup variations.

Our findings align with those of Gündoğar et al.^8^, who also reported no significant difference in postoperative pain among different irrigation activation systems. Similarly, Hanafy et al.^35^ observed higher pain scores with XP-endo Finisher, though the differences were not statistically significant. In contrast, other investigations such as Leoni et al.^31^ and Bao et al.^20^ demonstrated superior cleaning and biofilm removal with activation devices, which could potentially support improved patient outcomes. This inconsistency across studies highlights the complex nature of postoperative pain, where enhanced cleaning efficacy does not always translate into perceivable differences in patient discomfort.

Overall, the lack of statistical significance in this study underscores the need for further research with larger sample sizes and longer follow-up periods. Future investigations should also incorporate additional variables such as operator technique, canal anatomy, and patient psychological factors to better elucidate the clinical relevance of irrigation activation systems in reducing postoperative pain.

One of the limitations of this study is the relatively small sample size, which may affect the generalizability of the findings. A larger sample with diverse patient demographics would provide more robust statistical power and allow for better interpretation of the results. Additionally, the study only assessed post-operative pain, while other important clinical outcomes, such as long-term healing and success rates, were not evaluated. Future research should incorporate follow-up periods beyond two weeks to assess the sustained effects of different irrigation activation techniques on treatment success.

Another limitation is the use of a single pain assessment method (VAS scale), which, while widely accepted, remains subjective. Incorporating objective biomarkers of inflammation or using additional pain scales could enhance the reliability of pain assessment. Moreover, the study did not consider factors such as preoperative anxiety, occlusal forces, or variations in operator technique, which may influence post-operative pain. Future studies should explore these variables and evaluate the efficacy of irrigation activation methods in complex cases, such as curved canals or retreatment cases, to provide a more comprehensive understanding of their clinical impact.

## Conclusions

Within the limitations of this research, it was determined that none of the irrigation methods examined fully eliminated post-operative pain. However, the severity of post-operative pain diminished gradually across all groups as time progressed. Although between-group differences were not statistically significant, the findings suggest that both Group II and Group III may provide better post-operative comfort than conventional needle irrigation. Additionally, gender differences were observed, with female participants reporting higher pain scores. Further studies of larger sample sizes are recommended to confirm these findings.

## Data Availability

The datasets used and/or analysed during the current study are available from the corresponding author on reasonable request.
